# Numerical Investigation of the Performance of a Proton Exchange Membrane Water Electrolyzer under Various Outlet Manifold Structure Conditions

**DOI:** 10.3390/ma17153694

**Published:** 2024-07-26

**Authors:** Guobin Zhang, Zhiguo Qu

**Affiliations:** MOE Key Laboratory of Thermo-Fluid Science and Engineering, School of Energy and Power Engineering, Xi’an Jiaotong University, Xi’an 710049, China; zhangguobin@xjtu.edu.cn

**Keywords:** water electrolyzer, manifold structure, liquid water and gas flow, VOF, 3D electrolyzer model

## Abstract

The oxygen discharge process significantly affects the electrochemical performance of a proton exchange membrane water electrolyzer (PEMWE), which requires an optimal structure of the flow field implemented in the bipolar plate (BP) component. In this study, we numerically investigated the two-phase (liquid water and oxygen) flow in the PEMWE’s channel region with different outlet manifold structures utilizing the volume of fluid (VOF) model. Then, the oxygen volume fraction at the liquid/gas diffusion layer (L/GDL) surface, i.e., the interface of the channel and L/GDL, obtained by the liquid water and oxygen flow model was incorporated into a three-dimensional (3D) PEMWE model, which made it possible to predict the influence of the outlet manifold structure on the multiple transfers inside the whole electrolyzer as well as the electrochemical performance. The results indicate that the existence of oxygen in the flow field significantly decreased the electrolyzer voltage at a fixed operation current density and deteriorated the uniform distribution of the oxygen amount, current density (corresponding to the electrochemical reaction rate) and temperature in the membrane electrode assembly (MEA), indicating that the rapid oxygen removal from the flow field is preferred in the operation of the electrolyzer. Moreover, slight increases in the width of the outlet manifold were helpful in relieving the oxygen accumulation in the anode CL and, hence, improved the electrolyzer performance with more uniform distribution characteristics.

## 1. Introduction

Against the background of a worldwide demand for carbon neutrality by 2050–2060, hydrogen energy has received considerable attention because of its merits: zero emissions, high calorific value, etc. [1]. Moreover, hydrogen can be more conveniently stored than electricity, which plays a vital role in mitigating the fluctuations during the utilization of renewable energies, e.g., solar energy [2]. Consequently, converting the extra renewable energy that cannot be transmitted directly to the grid into hydrogen through a water electrolyzer is likely to significantly promote the building of renewable energy systems in the future [3]. Currently, the alkaline water electrolyzer (AWE) is being commercialized [4], but it suffers from a low hydrogen production rate and purity [5]. In comparison, the proton exchange membrane water electrolyzer (PEMWE) is receiving considerable attention due to its high efficiency and hydrogen purity and fast response. However, its development is still largely hindered by the high cost, and its performance is also unsatisfactory [6]. Specifically, the state-of-the-art operation current density of PEMWEs is about 2–3 A cm^−2^ [7], which should be increased to 10 A cm^−2^, leading to a higher hydrogen production rate and thus satisfying the commercialization requirements [8]. Meanwhile, it also contributes to a cost reduction for a fixed amount of hydrogen.

During the operation of PEMWEs, multiple physics processes occur simultaneously, including the two-phase flow (liquid water and gas: hydrogen and oxygen), electron and proton conduction, heat transfer and electrochemical reactions, and these processes range from micro-scale to meso-scale to macro-scale inside the PEMWE unit [9]. Optimizing these physical processes properly is of great importance in increasing the performance of PEMWEs. In general, the performance is highly dependent on the structural design and optimization of PEMWEs [10], which are, however, extremely complicated due to their multi-scale and multi-physics characteristics. 

Obviously, the structure of a PEMWE is mainly determined by the flow field configuration embedded in the bipolar plate (BP) component, which determines the water supply, product gas discharge, waste heat removal and the current collection from the liquid/gas diffusion layer (L/GDL) component [11]. Considering that the hydrogen evolution reaction (HER) rate in the cathode CL and the hydrogen transport rate are both much faster than the oxygen evolution reaction (OER) rate and oxygen transport rate, respectively, the oxygen discharge process on the anode side should receive more attention. Moreover, the anode flow field configuration and channel structure have a determinative influence on the oxygen discharge process [12], and insufficient oxygen removal will hinder the water supply and cover the electrochemical reaction site in CL [13], thereby reducing the performance of the PEMWE. 

Currently, a transparent PEMWE coupled with a high-speed camera is the most widely used configuration to observe the oxygen dynamic flow in channels [14]. Recently, Li et al. [15] studied the influence of the operation conditions on oxygen bubble evolution, such as nucleation, growth and detachment from the L/GDL, and they concluded that the influence of temperature on the oxygen growth rate is very significant, but the flow rate has little influence. Panchenko et al. [16] experimentally observed the two-phase flow dynamics in an electrolier with a stoichiometric ratio ranging from 95 to 1037 and found that about 37% and 63% of the pores in the PTL contribute to the transportation of liquid water and gas, respectively. Recently, Yuan et al. [17] conducted a comprehensive review with regard to the oxygen bubble evolution in PEMWEs, which sheds light on the investigation of the oxygen discharge process. 

So far, many efforts have been made to optimize the anode channel structure to enhance oxygen discharge. Toghyani et al. [18] compared the novel metal foam flow field with conventional flow fields in a PEMWE utilizing a 3D model and pointed out that the utilization of metal foam promotes the distribution of the electrochemical reaction rate (i.e., current density distribution) and improves the performance of the electrolyzer. Very recently, Wu et al. [19] proposed a novel dual-layer channel design concept that enhances the oxygen discharge capacity and contains a degassing layer with a larger cross-sectional area on the top conventional channel configuration. Su et al. [20] proposed a novel flow field that combines the structural characteristics of cross-finger, serpentine and cross flow fields, which strikes a good balance between the improvement in performance and the increase in the unfavorable pressure drop. However, the influence of the manifold structure on the oxygen bubble discharge process together with the electrochemical performance of the PEMWE has seldom been reported in the literature. Consequently, we first investigated the mechanism by which different outlet manifold structures are influenced via the novel integration of a two-phase channel model based on the volume of fluid method (VOF) and a three-dimensional (3D) PEMWE model. This work will further guide the design and optimization of bipolar plate (BP) components together with the flow field configuration.

## 2. Materials and Methods

**Figure 1:**
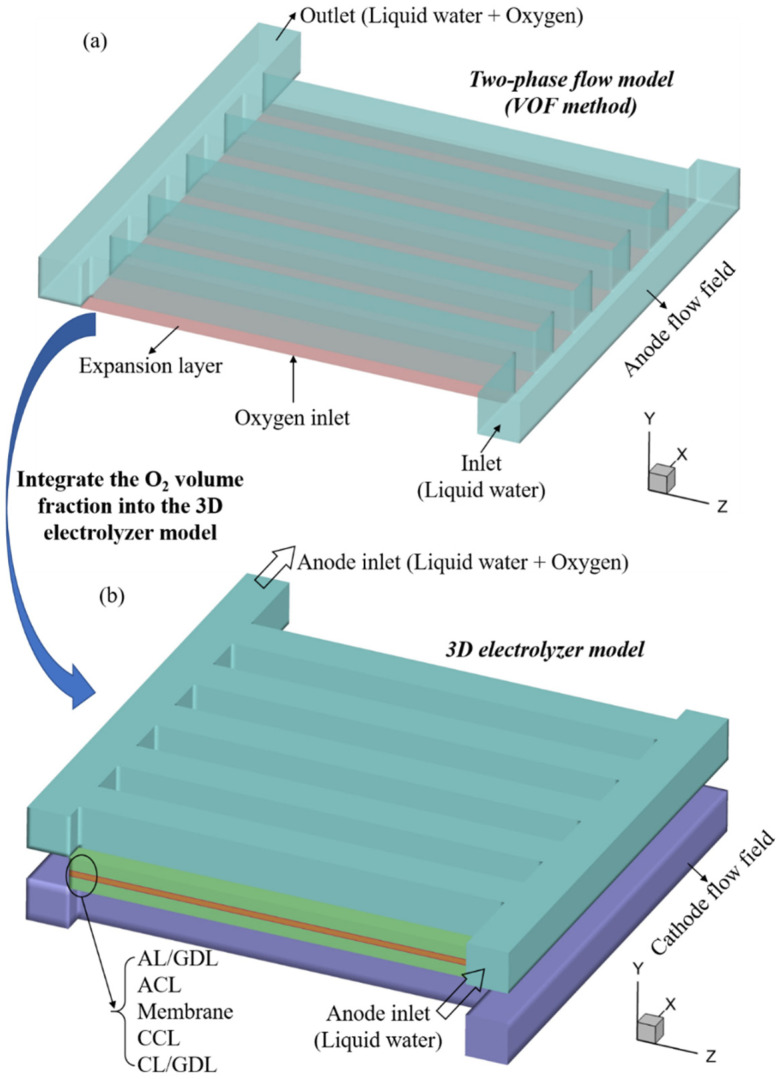
(**a**): Schematic of the two-phase flow model simulating the water (liquid state) and oxygen flow inside the anode flow field; (**b**): schematic of the 3D electrolyzer model for simulating the multiple transfers and electrochemical reactions in the entire PEMWE.

In this study, two models were developed, and their schematics are shown in Figure 1, which shows that one is a model for simulating the liquid water and oxygen flow in the anode flow field, and the other is a 3D electrolyzer model for simulating the multiple transfers and electrochemical reactions in the entire PEMWE.

The former is widely implemented to evaluate the oxygen discharge characteristics, but it is time-consuming and cannot incorporate the other physics processes in other components. As a consequence, it cannot directly evaluate the electrochemical performance of a PEMWE. On the contrary, the latter 3D electrolyzer model neglects the two-phase flow dynamics (simplifying it as single-phase flow by neglecting the oxygen) in the flow field, which results in its underestimation of the influence of oxygen discharge on the PEMWE performance. To solve this issue, the oxygen volume fraction obtained through the two-phase flow model was implemented into the 3D electrolyzer model to accurately predict the PEMWE performance, and the details will be presented in the next section. This integration method originated from the fact that neglecting the oxygen in the anode flow field will result in the 3D electrolyzer model being unable to accurately predict the influence of the flow field structure on the electrolyzer performance, although the 3D electrolyzer model can predict the electrolyzer’s I-V curves under various operation conditions [21,22].

### 2.1. Liquid Water and Oxygen Flow Model

Figure 1a shows the schematic of the liquid water and oxygen flow model, in which the flow field at the anode side was included in the computational domain. The volume of fluid (VOF) was utilized for modeling liquid water and oxygen flow, which is a common method for capturing the phase interface and has the capacity to simulate the realistic two-phase flow pattern in the anode flow field, e.g., the formation of bubbles due to the surface tension effect and the coalescence between bubbles. Note that an additional expansion layer (EL) was attached at the bottom surface of the flow field for consistency with the electrolyzer model, as shown in Figure 1b.

#### 2.1.1. Governing Equations

The governing equations are listed as below:

Mass and momentum conservation equations of the mixture of liquid water and oxygen:

(1) $$\frac{\partial \rho_{m}}{\partial t}+\nabla \cdot \left(\rho_{m}u_{m}\right)=0$$

(2) $$\frac{\partial \left(\rho_{m}u_{m}\right)}{\partial t}+\nabla \cdot \left(\rho_{m}u_{m}u_{m}\right)=-\nabla p+\nabla \cdot \left[\mu_{m}\left(\nabla u_{m}+\nabla u_{m}^{T}\right)\right]+\rho_{m}g+F_{s}$$

Oxygen phase (the second phase in modeling the two-phase flow) volume fraction equation:

(3) $$\frac{\partial s_{O_{2}}}{\partial t}+u\cdot \nabla s_{O_{2}}=0$$

And the liquid water phase (the primary phase in modeling the two-phase flow) volume fraction is calculated as follows:

(4) $$s_{l}=1-s_{O_{2}}$$

where u (m s^−1^), *p* (Pa), ρ (kg m^−3^), μ (Pa s), *t* (s), ***g*** (9.8 m s^−2^), ***F*_s_** (N m^−3^) and *s* are the velocity, pressure, density, viscosity, time, gravitational acceleration, source term with regard to surface tension and volume fraction of liquid water or oxygen, respectively. The subscripts m, O_2_ and l refer to the mixture, oxygen and liquid water, respectively. The source term of surface tension is expressed as follows:

(5) $$F_{s}=\sigma \frac{\bar{\rho }\kappa \nabla s_{O_{2}}}{0.5\left(\rho_{O_{2}}+\rho_{l}\right)}$$

(6) $$\kappa =\nabla \cdot \hat{n}=\nabla \cdot ({\hat{n}}_{w}\cos\theta_{w}+{\hat{t}}_{w}\sin\theta_{w})$$

where κ (m^−1^), σ (N m^−1^), $\hat{n}$ and $\theta_{w}$ (°) are the surface curvature between two phases (i.e., the primary phase of liquid water and the second phase of oxygen), surface tension coefficient, unit vector (${\hat{n}}_{w}$ normal and ${\hat{t}}_{w}$ tangential to the wall) and contact angle at the walls, respectively.

#### 2.1.2. Boundary Conditions

For this two-phase flow model, a constant velocity was set at the anode channel’s inlet and a constant pressure of 1.0 atm at the outlet. As for the oxygen, it entered the anode channel region from the bottom surface of the expansion layer, and the entrance velocity was set to be consistent with the realistic discharge velocity in the 3D electrolyzer model. At interface of the expansion layer and anode channel region, the type of boundary condition was set to ‘porous jump’ mainly because the expansion layer is in fact part of the L/GDL that is porous media in the PEMWE. And all surrounding surfaces were assigned the non-slip boundary condition.

### 2.2. 3D Electrolyzer Model

Figure 1b presents the 3D electrolyzer model’s computational domain, from which it can be seen that all basic components are included in the computational domain. In this model, it was assumed that the PEMWE operated in a steady state; the liquid water and oxygen flow was in a laminar regime; the L/GDL and CL were treated as homogenous porous media; the membrane could only transport protons and the proton conductivity in the membrane was constant mainly because the membrane was in a full hydration state. 

#### 2.2.1. Governing Equations

At the anode side, the water (liquid state) flow is governed by the mass conservation equation and momentum conservation equation:

(7) $$\frac{\partial }{\partial t}\left(\varepsilon s_{l}\rho_{l}\right)+\nabla \cdot \left(\rho_{l}u_{l}\right)=S_{\mathrm{ml}}$$

(8) $$\begin{array}{ll} \frac{\partial }{\partial t}\left(\frac{\rho_{l}u_{l}}{\varepsilon s_{l}}\right)+\nabla \cdot \left(\frac{\rho_{l}u_{l}u_{l}}{\varepsilon^{2}{s_{l}}^{2}}\right)= & -\nabla p_{l}+\mu_{l}\nabla \cdot \left[\nabla \left(\frac{u_{l}}{\varepsilon s_{l}}\right)+\nabla \left(\frac{u_{l}^{T}}{\varepsilon s_{l}}\right)\right]\\ & -\frac{2}{3}\mu_{l}\nabla \left[\nabla \cdot \left(\frac{u_{l}}{\varepsilon s_{l}}\right)\right]+S_{\mathrm{ul}} \end{array}$$

The oxygen pressure equation in the anode side (except the anode channel):

(9) $$\frac{\partial }{\partial t}\left(\rho_{g}\varepsilon s_{g}\right)+\nabla \cdot \left(\rho_{g}\frac{Kk_{g}}{\mu_{g}}\nabla p_{g}\right)=S_{p_{g}}$$

where ε, *K* (m^2^), *k*, $S_{\mathrm{ml}}$, $S_{p_{g}}$ (kg m^−3^ s^−1^) and $S_{\mathrm{ul}}$ (kg m^−2^ s^−2^) are the porosity, intrinsic permeability, relative permeability, source terms in mass, oxygen pressure and momentum equations, respectively.

At the cathode side, the gas mixture (hydrogen and water vapor) flow is also governed by the mass conservation equation and momentum conservation equation:

(10) $$\frac{\partial }{\partial t}\left(\varepsilon s_{g}\rho_{g}\right)+\nabla \cdot \left(\rho_{g}u_{g}\right)=S_{\mathrm{mg}}$$

(11) $$\begin{array}{ll} \frac{\partial }{\partial t}\left(\frac{\rho_{g}u_{g}}{\varepsilon s_{g}}\right)+\nabla \cdot \left(\frac{\rho_{g}u_{g}u_{g}}{\varepsilon^{2}{s_{g}}^{2}}\right)= & -\nabla p_{g}+\mu_{g}\nabla \cdot \left[\nabla \left(\frac{u_{g}}{\varepsilon s_{g}}\right)+\nabla \left(\frac{u_{g}^{T}}{\varepsilon s_{g}}\right)\right]\\ & -\frac{2}{3}\mu_{g}\nabla \left[\nabla \cdot \left(\frac{u_{g}}{\varepsilon s_{g}}\right)\right]+S_{\mathrm{ug}} \end{array}$$

Additionally, the gas species mass fraction equation is added in the cathode side:

(12) $$\frac{\partial }{\partial t}\left(\varepsilon s_{g}\rho_{g}Y_{i}\right)+\nabla \cdot \left(\rho_{g}u_{g}Y_{i}\right)=\nabla \cdot \left(\rho_{g}D_{i}^{\mathrm{eff}}\nabla Y_{i}\right)+S_{i}$$

The liquid water pressure equation in the cathode side (except the cathode channel) is as follows:

(13) $$\frac{\partial }{\partial t}\left(\rho_{l}\varepsilon s_{l}\right)+\nabla \cdot \left(\rho_{l}\frac{Kk_{l}}{\mu_{l}}\nabla p_{l}\right)=S_{l}$$

where $Y_{i}$, $D_{i}^{\mathrm{eff}}$ (m^2^ s^−1^), $S_{\mathrm{ug}}$ (kg m^−2^ s^−2^), $S_{\mathrm{mg}}$, $S_{i}$ (i: hydrogen or water vapor) and $S_{l}$ (kg m^−3^ s^−1^) are the gas species mass fraction, effective diffusion coefficient, source terms of momentum, mass, gas species and liquid water pressure equations, respectively. 

Oxygen saturation in anode L/GDL and CL and liquid water saturation in the cathode side are obtained through the semi-empirical equation describing the correlation of the capillary pressure and phase volume fraction (phase saturation) in porous media, i.e., the Leverett-J function [23]:

(14) $$p_{c}=p_{g}-p_{l}=\sigma \cos\theta {\left(\frac{\varepsilon }{K}\right)}^{0.5}J\left(s_{l}\right)$$

(15) $$J\left(s_{l}\right)=\left\{\begin{array}{cc} 1.42\left(1-s_{l}\right)-2.12{\left(1-s_{l}\right)}^{2}+1.26{\left(1-s_{l}\right)}^{3} & \theta <90^{\circ }\\ 1.42s_{l}-2.12{s_{l}}^{2}+1.26{s_{l}}^{3} & \theta >90^{\circ } \end{array}\right.$$

The electron and proton conductions in conjunction with the electrochemical reactions are described by electrical and ionic potential equations, in which the Butler–Volmer equations are added in the source terms [24]:

(16) $$0=\nabla \cdot \left(\kappa_{e}^{\mathrm{eff}}\nabla \varphi_{e}\right)+S_{e}$$

(17) $$0=\nabla \cdot \left(\kappa_{\mathrm{ion}}^{\mathrm{eff}}\nabla \varphi_{\mathrm{ion}}\right)+S_{\mathrm{ion}}$$

(18) $$S_{e}=\left\{\begin{array}{cc} -J_{a} & \mathrm{Anode}\;\mathrm{CL}\\ J_{c} & \mathrm{Cathode}\;\mathrm{CL} \end{array}\right.,\;S_{\mathrm{ion}}=\left\{\begin{array}{cc} J_{a} & \mathrm{Anode}\;\mathrm{CL}\\ -J_{c} & \mathrm{Cathode}\;\mathrm{CL} \end{array}\right.$$

(19) $$J_{a}=s_{l}^{2}J_{0,\;a}^{\mathrm{ref}}\left[\exp\left(\frac{\alpha_{a}F}{RT}\eta_{\mathrm{act},\;a}\right)-\exp\left(-\frac{(1-\alpha_{a})F}{RT}\eta_{\mathrm{act},\;a}\right)\right]$$

(20) $$J_{c}=J_{0,\;c}^{\mathrm{ref}}\left[\exp\left(\frac{\alpha_{c}F}{RT}\eta_{\mathrm{act},\;c}\right)-\exp\left(-\frac{(1-\alpha_{c})F}{RT}\eta_{\mathrm{act},\;c}\right)\right]$$

where $\varphi_{e}$ ($\varphi_{\mathrm{ion}}$, V), R (J mol^−1^ K^−1^), $\kappa_{e}^{\mathrm{eff}}$ ($\kappa_{\mathrm{ion}}^{\mathrm{eff}}$, S m^−1^), $J_{0,\;a}^{\mathrm{ref}}$ ($J_{0,\;c}^{\mathrm{ref}}$, A m^−3^), $\eta_{\mathrm{act},\;a}$ ($\eta_{\mathrm{act},\;c}$, V), $\alpha_{a}$ ($\alpha_{c}$), F (C mol^−1^) and T (K) are the electrical (ionic) potential, universal gas constant (8.314), effective conductivity, anode (cathode) current density at the reference state, activation overpotential, transfer coefficient, Faraday’s constant (96,487) and temperature, respectively.

The energy conservation equation is as follows:

(21) $$\begin{array}{c} \frac{\partial }{\partial t}\left(\varepsilon s_{l}\rho_{l}C_{p,\;l}T+\varepsilon s_{g}\rho_{g}C_{p,\;g}T\right)+\nabla \cdot \left(\varepsilon s_{l}\rho_{l}C_{p,\;l}u_{l}T+\varepsilon s_{g}\rho_{g}C_{p,\;g}u_{g}T\right)\\ =\nabla \cdot (k^{\mathrm{eff}}\nabla T)+S_{T} \end{array}$$

where $k^{\mathrm{eff}}$ (W m^−1^ K^−1^), $C_{p}$ (J mol^−1^ K^−1^) and subscripts g and l are effective heat conductivity, specific heat capacity and gas and liquid phases, respectively. 

#### 2.2.2. Boundary Conditions

The anode/cathode outlet pressure was set to a constant of 1.0 atm. The cathode inlet was closed (‘wall’ boundary condition). The anode inlet’s flow rate ($m_{a}$, kg m^−2^ s^−1^) is related to the operation current density (I, A m^−2^), stoichiometry ratio (ξ, 55 in this work), activation area ($A_{\mathrm{act}}^{a}$, m^2^) and anode inlet area ($A_{\mathrm{in}}^{a}$, m^2^):

(22) $$m_{a}=\frac{M_{H_{2}O}\xi IA_{\mathrm{act}}^{a}}{2FA_{\mathrm{in}}^{a}}$$

where $M_{H_{2}O}$ (kg mol^−1^) is the water’s molar mass. The oxygen pressure value at the L/GDL surface in Equation (9) is calculated as below:

(23) $$p_{g}=p_{l}+p_{c}=p_{l}+\sigma \cos\theta {\left(\frac{\varepsilon }{K}\right)}^{0.5}J\left(s_{O_{2}}\right)$$

This equation shows that the oxygen pressure is equal to the liquid water pressure when the oxygen amount in channel is zero. And the oxygen amount distribution (treated as 100 × 100 dataset) obtained through the VOF model is transferred into Equation (23) as the boundary condition of Equation (9). Furthermore, in order to improve the accuracy, it is preferable to further incorporate the simulated inhomogeneous current density corresponding to the inhomogeneous oxygen flow rate at the bottom surface of the EL into the VOF model and start a new round of integration, although this is very time-consuming. In fact, although the inhomogeneous current density influences the oxygen production to some extent, its influence on the oxygen distribution in the anode flow field is not significant, which can be proved by the simulation case in our previous studies [21,22]. As for the electrical potential equation, the operation current density (1.0 A cm^−2^) was set at the anode BP’s outer surface, and 0 V was assigned at the cathode BP. 

### 2.3. Numerical Implementation

In this study, both these two models were solved via ANSYS FLUENT 19.2, and the user defined function (UDF) was activated to incorporate the pressure equation, electron/proton conduction equation, electrochemical reactions and all the self-defined transport parameters and source terms. The model validation and grid independence test have been conducted in our previous works [21,22]. The operation condition parameters, geometry parameters and model parameters are given in detail in Table 1.

## 3. Results

### 3.1. Oxygen Discharge Characteristics in Anode Flow Field

**Figure 2:**
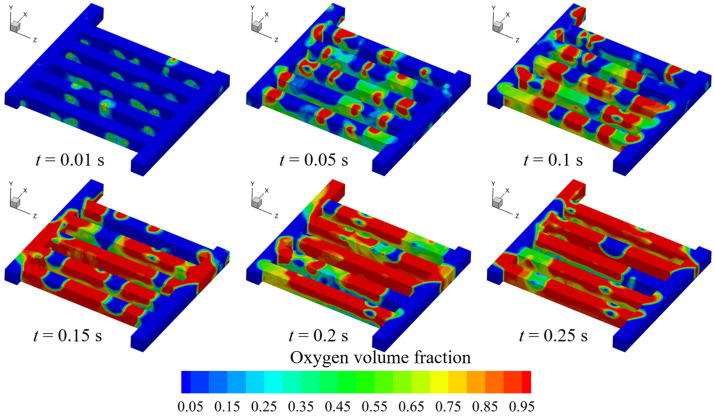
Transient oxygen volume fraction evolution process.

We numerically investigated the oxygen discharge process in anode channel region via the VOF model. Figure 2 shows the transient volume fraction evolution in the whole flow field. 

The figure shows that the oxygen amount increases gradually over time. At the initial moment (e.g., *t* = 0.01 s), there few bubbles are formed in the channels; as time goes on, more bubbles are formed in the channels (e.g., *t* = 0.05 s), and subsequently these bubbles start to coalesce (*t* = 0.1 s), forming a slug flow pattern. Moreover, it is interesting to find that the oxygen distribution in the whole flow field does not show significant variations when *t* > 0.15 s. However, it must be noted that the channel two-phase flow does not have an absolutely stable state, although the PEMEC can run in constant conditions.

**Figure 3:**
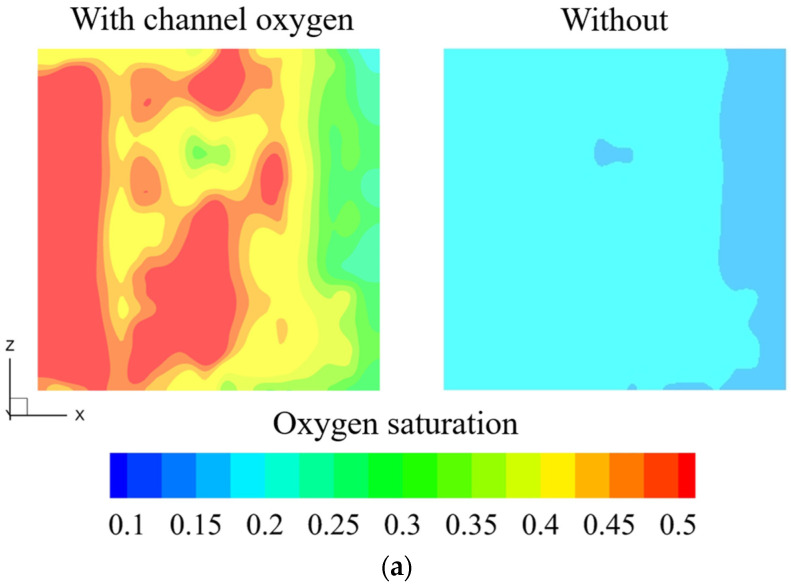

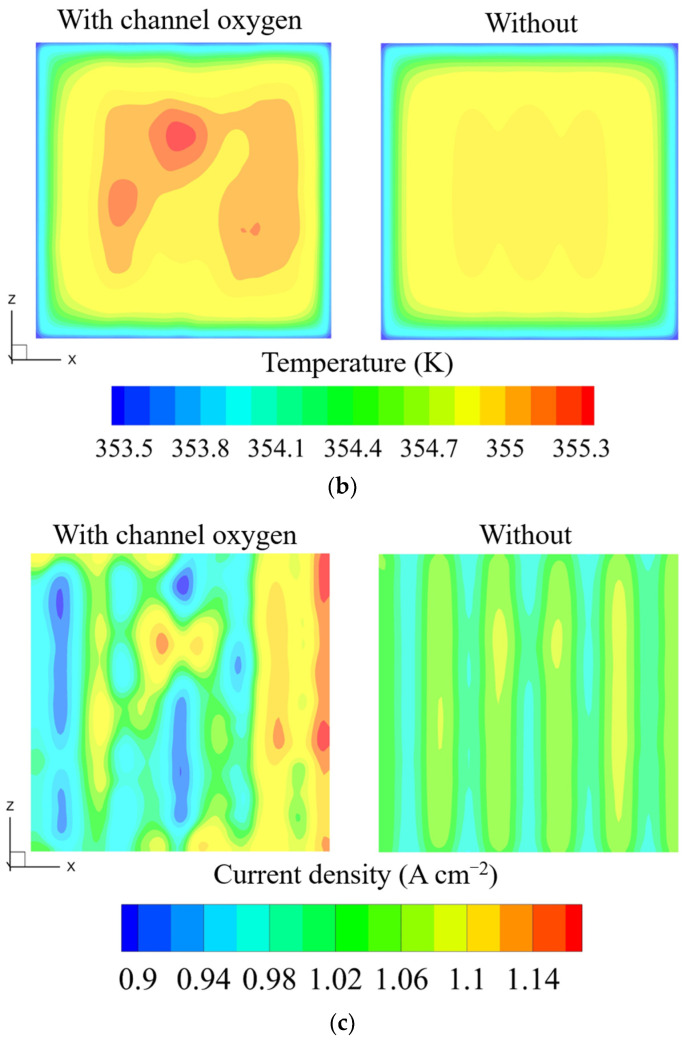
(**a**): Oxygen distribution contours in anode CL; (**b**): temperature distribution contours in anode CL; (**c**): distribution of membrane’s current density in the simulation cases with and without considering the channel oxygen (*I* = 1.0 A cm^−2^).

As mentioned in the previous section, we transferred the oxygen volume fraction (recognized as a 100 × 100 dataset) at the L/GDL surface (*t* = 0.15 s in Figure 2) into the 3D electrolyzer model to analyze the effect of oxygen distribution in channel region on the multiple transfers as well as the electrochemical reactions inside the MEA. Note that there is no steady state for the channel two-phase flow. For better comparison, the oxygen distribution in the anode flow field at a fixed time was selected. Figure 3 presents the oxygen, temperature and current density distribution contours with and without considering the channel oxygen.

Clearly, the channel oxygen significantly worsens the uniform distribution characteristics. Specifically, the channel oxygen largely hinders the oxygen discharge from the CL and L/GDL into the channel, and the highest oxygen volume fraction area corresponds to the oxygen accumulation zone (i.e., the oxygen volume fraction distribution at *t* = 0.15 s in Figure 2).

Furthermore, the non-uniform oxygen distribution induces the non-uniform temperature (in CL) and membrane’s current density distributions. In fact, the working voltage of the PEMWE increases from 0.77 V to 0.82 V considering the effect of channel oxygen. Consequently, it also contributes to the increase in temperature in the anode CL (Figure 3b). The simulation results presented in Figure 3 demonstrate that the influence of channel oxygen in the anode flow field is far from negligible [14,22]. It should be noted that the current (i.e., the product of current density and cross-sectional area) is constant (except in the CL component due to the electrochemical reactions) along the through-plane direction of the electrolyzer because these components are connected in series, but the local current density distribution is not identical as it is influenced by the local temperature, oxygen volume fraction, etc. Therefore, as seen in Figure 3c, the current density distribution in the simulation cases considering the oxygen in the channel is much more non-uniform than that without the channel oxygen, but the current and average current density (i.e., 1.0 A cm^−2^) is the same as the boundary value. 

### 3.2. Influence of Outlet Manifold Structures 

**Figure 4:**
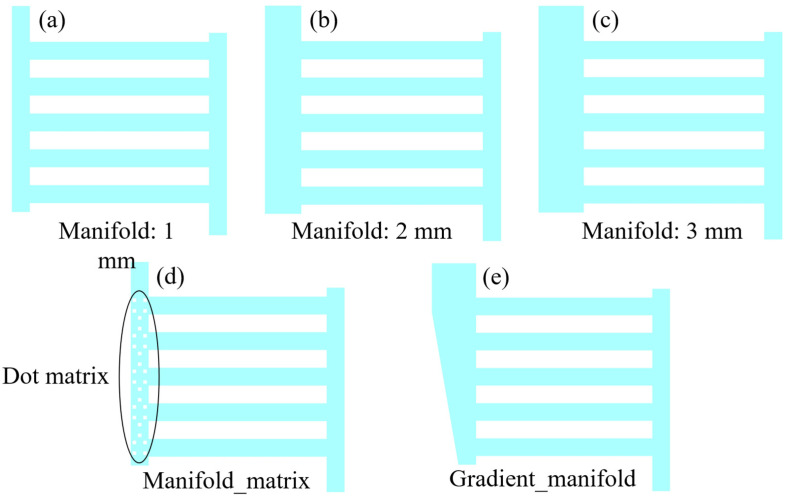
Schematic of the anode flow field with five different outlet manifold structures, (**a**–**c**): outlet manifold widths of 1, 2 and 3 mm, respectively; (**d**): inserting a dot matrix into the outlet manifold; (**e**): variable outlet manifold width gradually increasing from 1 mm to 2 mm.

Figure 4 presents the schematic of the anode flow field with five different outlet manifold structures investigated in this modeling work, namely, different outlet manifold widths of 1, 2 and 3 mm; inserting a matrix into the outlet manifold and a variable outlet manifold width gradually increasing from 1 mm to 2 mm.

**Figure 5:**
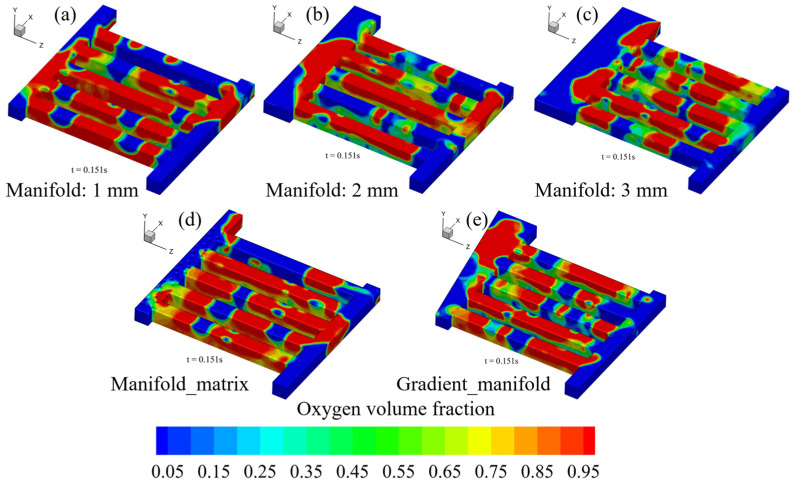
Oxygen amount distribution in flow fields with five different outlet manifold structures, (**a**–**c**): outlet manifold widths of 1, 2 and 3 mm, respectively; (**d**): inserting a dot matrix into the outlet manifold; (**e**): variable outlet manifold width gradually increasing from 1 mm to 2 mm.

Figure 5 presents the oxygen volume fraction distribution in these five different anode flow fields simulated via the VOF model (*t* = 0.15 s).

**Figure 6:**
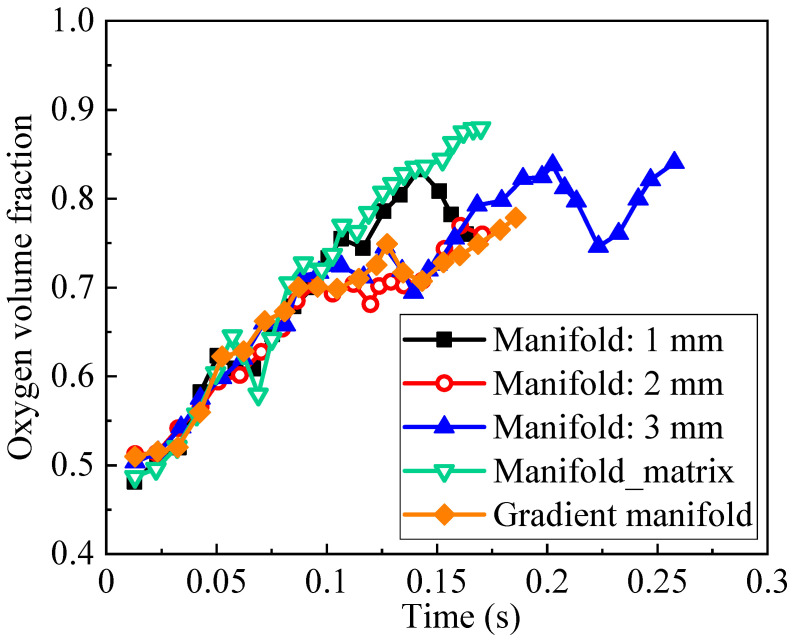
Average oxygen amount in flow fields with five different outlet manifold structures.

The figure shows that the oxygen volume fraction distribution characteristics do not show significant differences. However, increasing the outlet manifold width from 1 to 2 mm promotes the oxygen discharge in the channel region, but inserting a dot matrix into the outlet manifold zone blocks the oxygen discharge process. Moreover, the transient evolution of the average oxygen amount in the entire flow field presented in Figure 6 proves the findings. It should be noted that the channel two-phase flow does not have an absolutely steady state, and the oxygen volume fraction and corresponding distribution continuously vary even in the constant operation conditions. Even so, the simulation results shown in Figure 6 are sufficient to show the difference of these five structures in the oxygen discharge process, and the simulation results indicate that the variable outlet manifold width is not effective.

**Figure 7:**
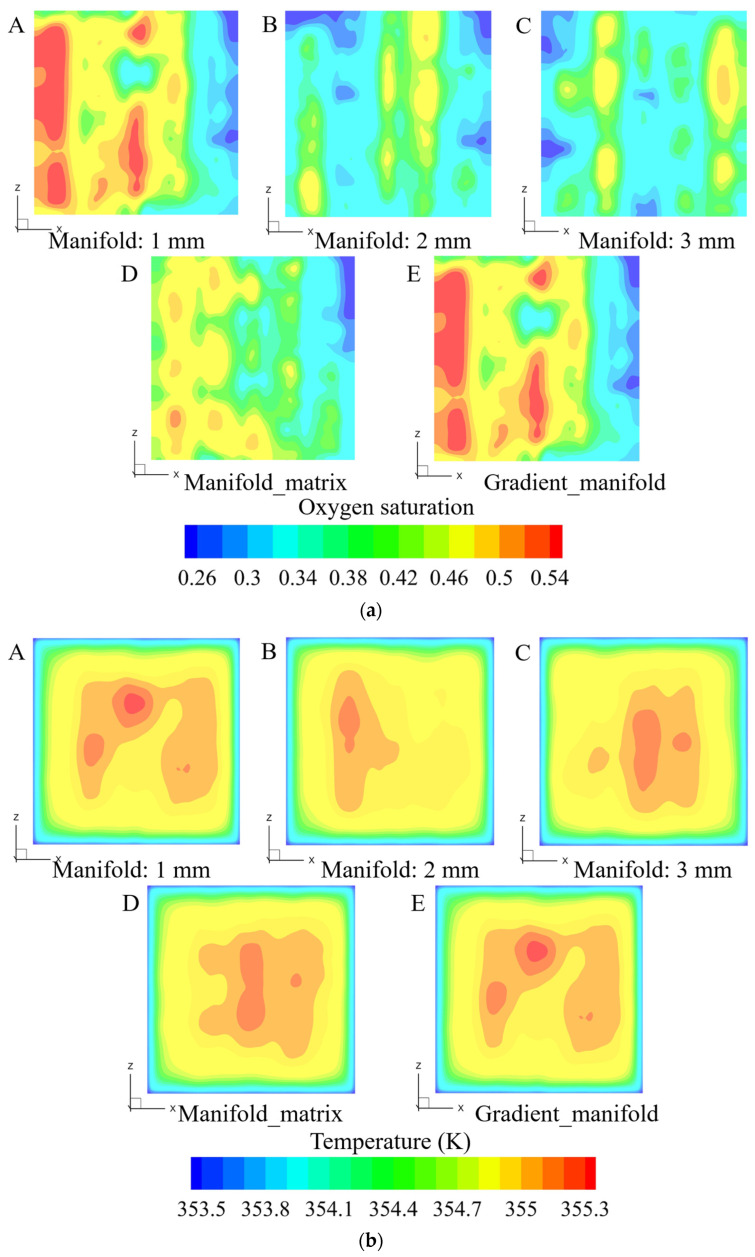

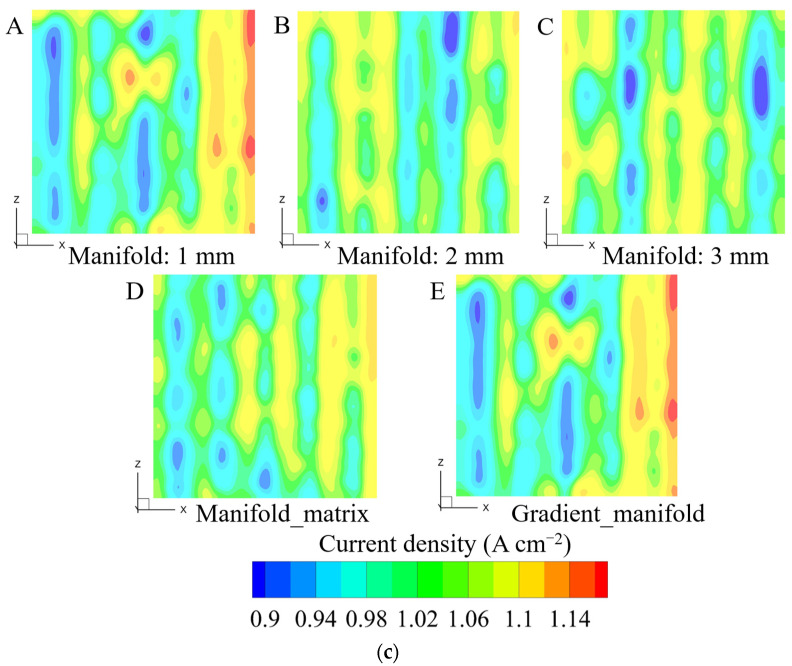
Variable distribution characteristics in the MEA of PEMWEs with five different outlet manifold structures in the anode flow field, (**a**): oxygen saturation distribution contours in anode CL; (**b**): temperature distribution contours in anode CL; (**c**): current density contours in membrane. (**A**–**C**): outlet manifold widths of 1, 2 and 3 mm, respectively; (**D**): inserting a dot matrix into the outlet manifold; (**E**): variable outlet manifold width gradually increasing from 1 mm to 2 mm.

Figure 7 presents the distribution contours of the oxygen saturation, temperature (in CL) and membrane’s current density in the PEMWEs with five different outlet manifold structures.

**Figure 8:**
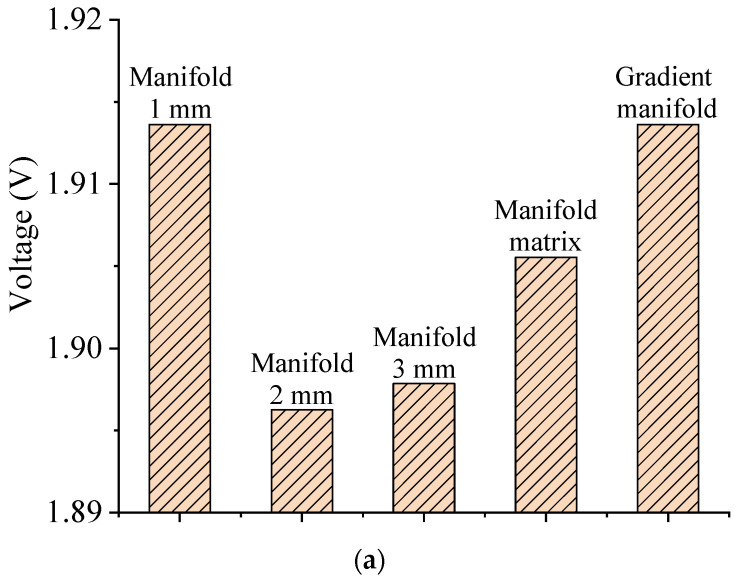

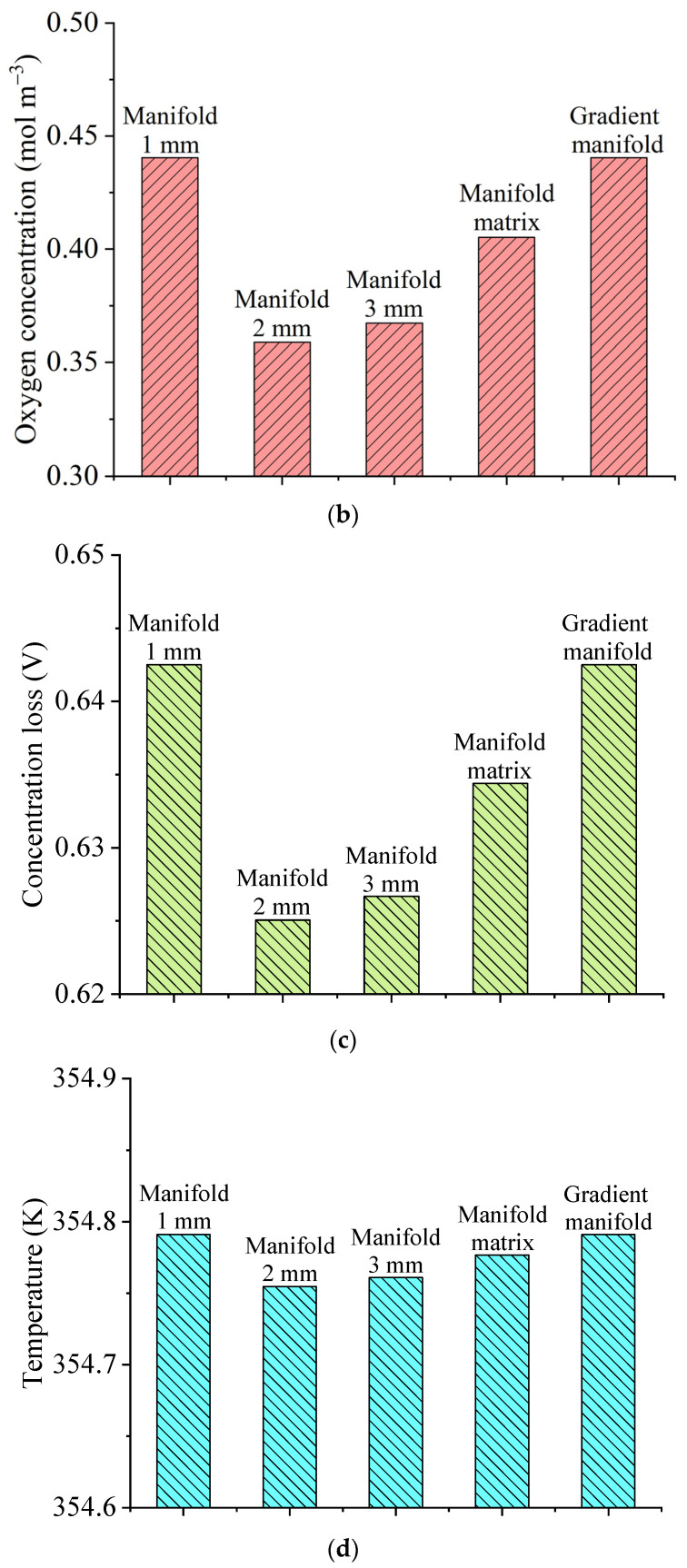
(**a**): Operation voltage; (**b**): average oxygen concentration in anode CL; (**c**): average concentration loss in anode CL; (**d**): average temperature in anode CL (1.0 A cm^−2^).

The figure shows that increasing the outlet manifold width promotes the uniform distribution characteristics. Furthermore, the outlet manifold width of 2 mm corresponds to the best PEMWE performance, as shown in Figure 8a, which is mainly a result of the PEMWE benefitting from the reduced oxygen concentration and concentration loss in anode CL, as presented in Figure 8b and Figure 8c, respectively.

Additionally, taking the outlet manifold width of 1 mm as the base case, all these four cases with optimized outlet manifold structures improve the PEMWE performance to different extents due to lower concentration loss. And the improved PEMWE performance also accompanies a reduction in average temperature, as shown in Figure 8d, which is mainly because the lower operation voltage leads to a lower amount of waste heat being generated.

## 4. Conclusions

In this work, we numerically investigated the influence mechanism of different outlet manifold structures through the integration of a liquid water and oxygen flow model via the VOF method and a 3D PEMWE model. The former focused on the simulation of liquid water and oxygen flow in the flow field, and the latter was capable of simulating the multiple ‘gas–liquid–heat–electron–proton’ transfers as well as the electrochemical reactions in all the basic components. The integration of these two models was implemented by incorporating the distributed oxygen volume fraction obtained by the two-phase flow model into the 3D electrolyzer model. For better integration, an extra expansion layer was added to the bottom of the anode flow field. Through this novel modeling method, we pointed out that the incorporation of oxygen distribution in the channel region was of significant importance in accurately predicting the electrochemical performance of a PEMWE together with the distribution contours of oxygen, membrane’s current density and CL’s temperature in the MEA. In addition, the simulation results showed that increasing the outlet manifold width from 1 mm to 2 mm significantly improved the PEMWE performance, which was mainly because this design enhanced the oxygen discharge process and hence decreased the concentration loss. Moreover, the improved electrochemical performance, i.e., lower operation voltage at fixed current density, decreased the generation of waste heat and hence reduced the temperature inside the MEA. And the other four outlet manifold structures, i.e., increasing the outlet manifold width from 1 mm to 3 mm, inserting a dot matrix into the outlet manifold and a variable outlet manifold width gradually increasing from 1 mm to 2 mm, also improved the PEMWE performance to different extents, which was also due to the enhancement of the oxygen discharge process. Overall, it can be concluded that the oxygen discharge characteristics in the anode flow field must be considered in BP and flow field structure design and optimization, and the simulation method proposed in this study was a powerful tool in this regard. 

## Figures and Tables

**Table 1:** Operation conditions, geometry parameters and model parameters.

Parameters	Values
Working pressure, current density, temperature	1.0 atm, 1.0 A cm^−2^, 353.15 K
MEA area	1.0 cm^2^
Membrane, CL, L/GDL thickness	127, 10, 300 μm
Channel/BP width, height	1.0, 1.0 mm
Porosity: CL, anode L/GDL, cathode L/GDL	0.3, 0.75, 0.6
Reference hydrogen and oxygen concentrations	33.5, 33.5 mol m^−3^
Anode and cathode reference exchange current densities	1.0 × 10^9^, 8.0 × 10^4^ A m^−3^
Transfer coefficient	0.5
Electrical conductivity: anode L/GDL, cathode L/GDL, anode CL, cathode CL, BP	20,000, 2000, 1000, 5000, 20,000
Membrane’s equivalent weight and density	1.1 kg mol^−1^, 1980 kg m^−3^
Electrolyte volume fraction in CLs	Anode: 0.2, cathode: 0.3
Contact angles: anode L/GDL, cathode L/GDL, anode CL, cathode CL, L/GDL surface, BP surface	70°, 120°, 80°, 100°, 120°, 90°
Permeabilities: anode L/GDL, cathode L/GDL, anode CL, cathode CL, membrane	4.9 × 10^−11^, 2.0 × 10^−12^, 2.0 × 10^−12^, 1.0 × 10^−13^, 2.0 × 10^−20^
Water phase change coefficient	100 s^−1^

## Data Availability

The raw data supporting the conclusions of this article will be made available by the authors on request.
